# Associations between COVID-19 pandemic impact, dimensions of behavior and eating disorders: a longitudinal UK-based study

**DOI:** 10.1192/j.eurpsy.2022.267

**Published:** 2022-09-01

**Authors:** K. Ioannidis

**Affiliations:** Cambridge and Peterborough NHS Foundation Trust, S3 Eating Disorders, Cambridge, United Kingdom

**Keywords:** pandemic, Eating Disorders, Covid-19, Impulsivity

## Abstract

**Introduction:**

There is growing concern about how people with eating disorders are impacted by the widespread societal restructuring during the COVID-19 crisis.

**Objectives:**

We aimed to examine how factors relating to the impact of the pandemic associate with eating disorders and quantify this relationship while adjusting for concurrent and longitudinal parameters of risk.

**Methods:**

We gathered demographic, behavioral and clinical data pre- and mid-pandemic as well as childhood trauma history from a longitudinal online survey of 489 adults (mean age 23.4 years) recruited from the Neuroscience in Psychiatry Network (NSPN). Using pre-pandemic (T1) and concurrent (T2) data we aimed to predict eating disorders at mid-pandemic (T2). We deployed hierarchical generalized logistic regression to ascertain the strength of longitudinal and concurrent associations.

**Results:**

Pre-pandemic eating disorder scores strongly associated with concurrent eating disorder (z=5.93). More conflict at home mid-pandemic (z=2.03), pre- (lower sensation seeking z=-2.58) and mid-pandemic (higher lack of perseverance z=2.33) impulsivity traits also associated with mid-pandemic eating disorder. Significant correlations between pandemic-related disrupted lifestyle and eating disorder psychopathology both pre- and mid-pandemic were observed.

**Conclusions:**

Conflict at home mid-pandemic and specific aspects of impulsiveness significantly associated with concurrent eating disorder when adjusted for pre-pandemic eating disorder symptoms, baseline demographics, behavioral traits, history of traumatic experiences and concurrent psychopathology. These results provide insight into the struggles of those suffering with eating disorders during the COVID-19 pandemic and highlight the importance of impulsiveness traits and the immediate family environment in their experience of illness during the pandemic.

**Disclosure:**

No significant relationships.

